# Evaluation of flattening-filter-free and flattening filter dosimetric and radiobiological criteria for lung SBRT: A volume-based analysis

**DOI:** 10.3389/fonc.2023.1108142

**Published:** 2023-01-24

**Authors:** Junxiang Wu, Hongchang Song, Jie Li, Bin Tang, Fan Wu

**Affiliations:** ^1^ Radiation Oncology Key Laboratory of Sichuan Province, Sichuan Cancer Hospital & Institute, Sichuan Cancer Center, School of Medicine, University of Electronic Science and Technology of China, Chengdu, China; ^2^ Department of Oncology, Xichang People’s Hospital, Xichang, China

**Keywords:** SBRT, lung cancer, FFF beams, VMAT, NTCP

## Abstract

**Introduction:**

The use of volumetric modulated arc therapy (VMAT) with flattening-filter-free (FFF) beams is becoming more prevalent in lung cancer stereotactic body radiotherapy (SBRT). The aim in this study was to assess the impact of dosimetric and radiobiological differences between FFF and flattening filter (FF) beams for lung SBRT based on the target volume.

**Methods:**

A total of 198 lung stereotactic body radiation therapy treatment plans with FFF beams and FF beams were retrospectively selected for this study. For all plans, the prescribed dose was 50 Gy/5 fractions, and the dose volume histogram (DVH) for the target and organs at risk (OAR) and the normal tissue complication probability (NTCP) of the lung were recorded and compared. Moreover, monitor units (MUs), the beam on-time and the treatment time were evaluated.

**Results:**

The study was performed following the Radiation Therapy Oncology Group (RTOG) 0813 and 0915 protocols. No significant differences in D_90_, coverage rate (CR) or conformity index (CI) of the target were observed between FFF beams and FF beams (*p*>0.05). The D_2_, R_50%_ and gradient index (GI) for the target improved with FFF beams compared with FF beams (*p*<0.05). FFF beams also significantly reduced the dose for the lung, heart, spinal cord, esophagus and NTCP of the lung (*p*<0.05), compared with FF beams. However, there was no significant difference in sparing of the trachea (*p*>0.05). The mean MUs, beam on-time and treatment time were 1871 ± 278 MUs, 3.2 ± 0.2 min and 3.9 ± 0.3 min for FFF beams, and 1890 ± 260 MUs, 4.2 ± 0.3 min and 4.8 ± 0.4 min for FF beams, respectively.

**Discussion:**

The FFF beam technique for lung SBRT with VMAT results in a better dose fall-off, better dose-sparing of OAR, lower NTCP of the lung and a shorter beam on-time compared with the FF beam technique. Additionally, the improvement in target and OAR-sparing for FFF beams was increased with increasing target volume.

## Introduction

Among Chinese men, lung cancer is the most common cancer, accounting for about 24.6% of all new cancer cases, according to data released in 2022 by the Chinese National Cancer Center (1). The use of stereotactic body radiation therapy (SBRT) has become the standard treatment for medically inoperable, early stage, non-small-cell lung cancer (NSCLC) (2–4). Compared to conventional radiation therapy, SBRT has the major feature of delivering large doses, while minimizing the dose to the organs at risk (OAR) (5–7). However, with the delivery of large doses, which results in a prolonged beam on-time, it could impact patient safety, such as patient motion, and the succeeding risk of dose uncertainties increases. The crucial challenges of SBRT concern safety, accuracy, and speed.

Due to advances in technology, there has been growing interest in the clinical use of flattening-filter-free (FFF) beams to deliver lung SBRT treatment (8–10). The FFF beam has had the flattening filter removed, such that the beam is not flat, as flatness is not necessary for volumetric modulated arc therapy (VMAT). The main characteristics of FFF beams are the high dose rate (1400 monitor units per minute for 6 MV photon beam), a cone-shaped dose profile (11), reduced peripheral dose outside of the beam (12) and an increased superficial dose. The most notable results of FFF beams are the large reduction in beam on-time, increased clinical efficiency, a better target dose coverage and OAR-sparing (8, 10, 13, 14).

With these advantages of FFF beams, many researchers have studied the dosimetry of FFF beams and FF beams in lung SBRT (8, 15, 16). However, according to a previous paper (17) and our experience, the difference in plan quality between FFF beams and FF beams is dependent on PTV. Only Reggiori et al. (17) have compared FFF beams with FF beams based on PTV for liver SBRT. To the best of our knowledge, there have been few studies evaluating the dosimetric and radiobiological differences between FFF beams and FF beams for lung SBRT based on PTV. Treatment plan evaluation metrics for lung SBRT were recommended by the Radiation Therapy Oncology Group (RTOG) 0813 and 0915 protocols; these include R_50%_, the gradient index (GI), the conformity index (CI), and the coverage rate (CR) for PTV, and dose volume limits for OAR (18, 19). Furthermore, the normal tissue complication probability (NTCP) for the lung was evaluated between FFF beams and FF beams. The patients were divided into four groups according to the 0813 and 0915 protocols, as Group1 (3.8 cm^3^≤PTV<7.4 cm^3^), Group 2(7.4 cm^3^≤PTV<13.2 cm^3^), Group 3(13.2 cm^3^≤PTV<22.0 cm^3^) and Group 4(22.0 cm^3^≤PTV ≤ 34.0 cm^3^). In this work, we retrospectively compared the dosimetry and NTCP of 175 lung SBRT patients, using VMAT with FFF beams and FF beams dependent on PTV.

## Materials and methods

### Patients

175 patient with 198 pulmonary lesions (198 independent isocentre) treated with SBRT using either FFF or FF beams during June 2021 to March 2022 were included in the study. This retrospective study was approved by the Ethics Committee of our hospital. Patient characteristics are summarized in **Table 1**. The patients were immobilized in a supine position with their arms above their head. All patients received two computed tomography (CT) scans: a free-breathing CT scan and a four-dimensional CT scan (4DCT). CT scans with a 3 mm slice thickness were acquired with a 16-slice Brilliance Big Bore CT (Philips Medical Systems, Cleveland, OH, USA). All 10 phases of the 4DCT slices and respiratory motion data were transferred to MIM Maestro software (MIM Software Inc., Cleveland, OH, USA) using 4D Workflow, where the maximum intensity projection (MIP) images and average intensity projection (AIP) images were generated. Visible lesions on CT were used to determine the gross tumor volume (GTV) using the window/level settings on the lung (window/level ranges from 800 to 1600 HU or -600 to -750 HU), and the GTV and clinical target volume (CTV) was often considered to be equal (20, 21). CTVs from 4DCT were contoured on CT images of each respiratory phase (CTV0, CTV10 to CTV90) and added together to define the internal target volume (ITV). The planning target volume (PTV) was generated by expanding 5 mm in three dimensions to ITV. The volume for the PTV range was from 5.07 to 33.49 cc (mean 14.63 ± 5.25 cc), and the detail of the PTV is shown in **Table 1**. The patients were stratified into four groups according to the RTOG 0813 protocol by the volume lesion: the first group included 7 patients with a PTV from 3.8 to 7.4 cm^3^, the second group included 83 patients with a PTV from 7.4 to 13.2 cm^3^, the third group included 91 patients with a PTV from 13.2 to 22.0 cm^3^, and the fourth group included 17 patients with a PTV from 22.0 to 34.0 cm^3^. The OAR of this study were contoured on the CT image, which included the lung, heart, spinal cord, esophagus and trachea.

**Table 1:** Patient characteristics of 175 patients with 198 pulmonary lesions.

Patients			
Sex		Tumors site	
Male	115 (65.7%)	Left lung	100 (50.5%)
Female	60 (34.3%)	Right lung	98 (49.5%)
Age		Peripheral	181 (91.4%)
≤50	60 (34.3%)	Central	17 (8.6%)
>50	115 (65.7%)	Primary tumors	
PTV volume (cc)		Lung	135 (77.1%)
Group1 (3.8 ≤ PTV< 7.4)	7 (3.5%)	Liver	25 (14.3%)
Group2 (7.4 ≤ PTV< 13.2)	83 (41.9%)	Stomach	5 (2.9%)
Group3 (13.2 ≤ PTV< 22.0)	91 (46.0%)	Breast	6 (3.4%)
Group4 (22.0 ≤ PTV ≤ 34.0)	17 (8.6%)	Unknown	4 (2.3%)
Originally treated beams			
FFF beams	177 (89.4%)		
FF beams	21 (10.6%)		

### Treatment planning

**Figure 1:**
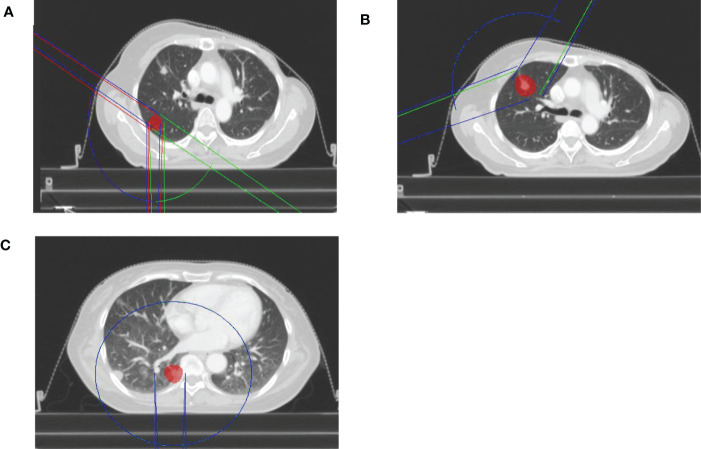
The arcs for right lung for FFF beams and FF beams. **(A)** the lower region of the lung. **(B)** the upper region of the lung. **(C)** the midline of the body.

All clinical treatment plans were generated using the Pinnacle TPS (version 9.10, Philips Radiation Oncology Systems, Fitchburg, WI, USA). Intensity modulation was performed using the direct machine parameter optimization (DMPO) algorithm. The collapsed cone (CC) algorithm was applied for final dose calculations, with a grid size of 2.5 mm. For each patient, two plans were created:1) a triple VMAT arc using FFF beams (VMAT_FFF_) with collimator 0° and couch 0°; and 2) a triple VAMT arc using FF beams (VMAT_FF_) with collimator 0° and couch 0°. The treatment beam was a non-coplanar6MV photon using the Infinity Linear Accelerator (Elekta AB, Sweden). The Infinity Linear Accelerator is equipped with a multileaf collimator, MLCi2, which has 40 leaf pairs of 0.5 cm thickness. The maximum dose rate of the FFF beams and FF beams was 1400 and 600 monitor units (MU)/min for 6 MV photons at the Elekta Infinity linear accelerator, respectively. VMAT_FFF_ and VMAT_FF_ plans were generated using 3 arcs; for example, when the target was in the right lung, the arcs were as follows: 1) located in the lower region of the lung with 3 partial arcs, the first of which was clockwise from 181˚ to 300˚, the second was counterclockwise from 300˚ to 181˚, and the third was clockwise from 120˚ to 179˚; 2) located in the upper region of the lung, with 3 partial arcs, the first of which was clockwise from 250˚ to 30˚, with the same range of angles for arc 2 and arc 3; and 3) located near the midline of the body with 3 full arcs, from 181˚ to 179˚.The arcs for right lung are presented in **Figure 1**.

The penalties and weights for the target and OAR, and the number of iterations were kept the same for the VMAT_FFF_ and VMAT_FF_ plans. The prescribed dose was 50 Gy in 5 fractions aimed to cover the PTV with 90% of the prescribed dose (D_90_).The dose constraints for the PTV were1) D_90_≥100% of the prescribed dose, and 2) D_2_ ≤ 125% of the prescribed dose. For the OAR, both the VMAT_FFF_ and VMAT_FF_ plans met the dose volume limits of the RTOG 0813/0915 protocols, as detailed in **Table 2**.

**Table 2:** Dose-volume constraints for OARs.

OARs	Dose volume parameters
Spinal cord	D_0.25cc_<22.5 Gy, D_0.5cc_<13.5 Gy, D_max_<30 Gy
Lung	D_1500cc_<12.5 Gy, D_1000cc_<13.5 Gy
Esophagus	D_5cc_<27.5 Gy
Heart	D_15cc_<32 Gy
Trachea	D_4cc_<18 Gy

### Evaluation of dosimetric data

The dosimetric quality of the treatments were measured using a dose volume histogram (DVH) metrics. For PTV, the target coverage (D_90_ and D_2_), the coverage rate (CR), the conformity index (CI), R_50%_ and the gradient index (GI) were reported (22–24).

CR was defined as:


(1) $$\text{CR}=\text{T}{\text{V}}_{\text{PV}}/\text{TV}$$

where TV_PV_ is the target volume covered by the prescription isodose and TV is the volume of the target.

CI was defined as:


(2) $$\text{CI}={\left(\text{T}{\text{V}}_{\text{PV}}\right)}^{2}/\left(\text{TV}\times \text{PV}\right)$$

where PV is the volume covered by the prescription isodose. CI values range between 0 and 1, and a CI close to 1 represents better conformity. GI was defined as the ratio of the volume of half the prescription isodose and the TV_PV._ R_50%_ was calculated using the RTOG 0813 protocol (18), and it was defined as the ratio of 50% prescription isodose volume to the PTV. Furthermore, dosimetric parameters were evaluated for the lung, spinal cord, esophagus, heart and trachea. The dose to the lung was evaluated using V_5_, V_20_, D_1000cc_, D_1500cc_ and the mean lung dose (MLD). D_max_, D_0.25cc_ and D_0.5cc_ of spinal cord were recorded. For the esophagus, heart and trachea, the D_max_, D_5cc_, D_1500cc_ and D_4cc_ were scored, respectively. Moreover, monitor units (MUs),the beam on-time (BOT) and the treatment time for the two plans were also recorded.

### Evaluation of NTCP data

The NTCP for ≥ grade 2 radiation pneumonitis (RP) of the lung was assessed for VMAT_FFF_ and VMAT_FF_ plans. For all patients, we exported DVHs of the lung to convert the physical dose into the biologically effective dose (BED) (25), using the LQ model and α/β-ratio of 3 Gy (26), as follows:


(3) $$\text{BED=D}\left(1+\frac{\text{d}}{\text{α/β}}\right)\;\;\;\;\;\;\;$$

in which the total dose (D) is the dose per fraction (d) multiplied by the number of fractions (n). The Lyman–Kutcher–Burman (LKB) model of NTCP (27) can be calculated from the EUD or V_x_ according to:


(4) $$\text{NTCP=}\frac{1}{\sqrt{2\pi }}\int_{-\infty }^{\text{t}}{\text{e}}^{\frac{-{\text{x}}^{2}}{2}}\text{dx}$$

With


$$\text{t=}\{\begin{array}{c} \frac{{\text{EUD-TD}}_{50}}{\text{m}\cdot {\text{TD}}_{50}}\text{ , for MLD}\\ \;\;\;\;\;\;\;\frac{{\text{V}}_{\text{x}}{\text{-V}}_{x50}}{\text{m}\cdot {\text{V}}_{x50}}{\text{ ,for V}}_{5}{\text{, and V}}_{20} \end{array}$$

In Equation (4), the MLD (BED-corrected) can be used instead of the EUD to calculate the NTCP, according to Seppenwoolde et al. (28). TD_50_, the mean dose for 50%of the NTCP, and m, a slope parameter (the steepness of the curve decreases with increasing m), were 20.8 Gy and 0.45, respectively, for the equation based on MLD, as proposed by Borst et al. (26). V_x50_ represents the V_x_ parameter for 50% of the NTCP. The parameters for the NTCP model are given in **Table 3**.

**Table 3:** Parameters used to calculate NTCP.

Type	m	TD_50_	V_x50_
MLD	0.45	20.8	–
V_5_	0.46	–	65.4
V_20_	0.50	–	30.6

Data were statistically analyzed using SPSS 19.0 software (IBM, New York, USA). A paired t-test(two-sided) was used for analyzing the dosimetric and radiobiological parameters of the PTV and OAR between VMAT_FFF_ and VMAT_FF_ plans with normal distribution. A value of *p*<0.05 was considered to be significant.

## Results

### Dosimetric evaluation of PTV

All plans were normalized to cover 90% of the PTV with the prescribed dose and met the planning objectives, as described in **Table 2**.A comparison of the PTV and OAR dosimetric parameters for the VMAT_FFF_ and VMAT_FF_ plans for all 198 lung SBRT plans(generated on 175 patients with 198 independent isocentre) is presented in **Table 4**. In general, the dose within the PTV was similar for the VMAT_FFF_ plan and the VMAT_FF_ plan. There was no significant difference in D_90_, CR and the CI of the PTV between the two plans (*p*>0.05). However, the D_2_, R_50%_ and GI of the VMAT_FFF_ plan were significantly lower than the VMAT_FF_ plan (*p*<0.05). D_2_ of the PTV was 55.30 ± 0.45 Gy for the VMAT_FFF_ plan and 55.90 ± 0.50 Gy for the VMAT_FF_ plan. R_50%_ of the PTV was 4.88 ± 0.61 for the VMAT_FFF_ plan and 4.98 ± 0.64 for the VMAT_FF_ plan. GI of the PTV was 5.21 ± 0.73 for the VMAT_FFF_ plan and 5.33 ± 0.78 for the VMAT_FF_ plan.

**Table 4:** Comparison of the dosimetric parameters (mean ± standard)of PTV and OARs.

	Parameters	VMAT_FFF_ (range)	VMAT_FF_ (range)	*p*
PTV	D_90_ (Gy)	50.0 ± 0.21 (50.0-50.12)	50.0 ± 0.21 (50.0-50.13)	0.35
	D_2_ (Gy)	55.30 ± 0.45 (54.52-56.42)	55.90 ± 0.50 (54.68-57.52)	<0.001
	CR	0.85 ± 0.02 (0.80-0.95)	0.86 ± 0.02 (0.79-0.92)	0.21
	CI	0.78 ± 0.06 (0.62-0.91)	0.78 ± 0.07 (0.63-0.92)	0.15
	R_50%_	4.88 ± 0.61 (3.29-6.72)	4.98 ± 0.64 (3.96-6.88)	<0.001
	GI	5.21 ± 0.73 (3.52-6.64)	5.33 ± 0.78 (3.55-6.91)	<0.001
Spinal cord	D_max_(Gy)	7.29 ± 4.37 (1.52-26.46)	7.44 ± 4.42 (1.63-26.55)	<0.001
	D_0.25cc_(Gy)	6.60 ± 3.85 (1.23-22.7)	6.81 ± 3.93 (1.3-22.75)	<0.001
	D_0.5cc_ (Gy)	6.37 ± 3.66 (1.05-21.62)	6.60 ± 3.88 (1.1-21.56)	<0.001
Esophagus	D_max_(Gy)	6.95 ± 4.43 (0.04-24.72)	7.08 ± 4.39 (0.05-23.94)	0.011
	D_5cc_ (Gy)	3.71 ± 2.67 (0.37-16.13)	3.81 ± 2.72 (0.44-15.74)	<0.001
Trachea	D_max_(Gy)	5.12 ± 5.37 (0.03-23.16)	5.20 ± 5.37 (0.03-23.77)	0.13
	D_4cc_(Gy)	3.11 ± 3.44 (0.01-13.81)	3.06 ± 3.44 (0.02-13.95)	0.25
Lung	D_1000cc_ (Gy)	1.21 ± 0.66 (0.08-4.10)	1.34 ± 0.93 (0.08-5.22)	<0.001
	D_1500cc_ (Gy)	0.51 ± 0.37 (0.05-2.44)	0.55 ± 0.40 (0.05-2.70)	<0.001
	MLD(Gy)	2.39 ± 0.78 (0.61-4.59)	2.47 ± 0.80 (0.63-4.68)	<0.001
	V_5_(%)	12.10 ± 3.89 (2.11-24.15)	13.04 ± 3.90 (2.42-24.35)	<0.001
	V_10_(%)	6.69 ± 2.47 (1.12-13.97)	7.02 ± 2.42 (1.21-14.86)	0.013
	V_20_(%)	3.01 ± 1.39 (0.14-6.96)	3.07 ± 1.35 (0.14-7.27)	0.016
Heart	D_1500cc_ (Gy)	5.48 ± 5.0 (0.06-36.48)	5.61 ± 5.05 (0.05-36.13)	<0.001

### Dosimetric evaluation of OAR

**Figure 2:**
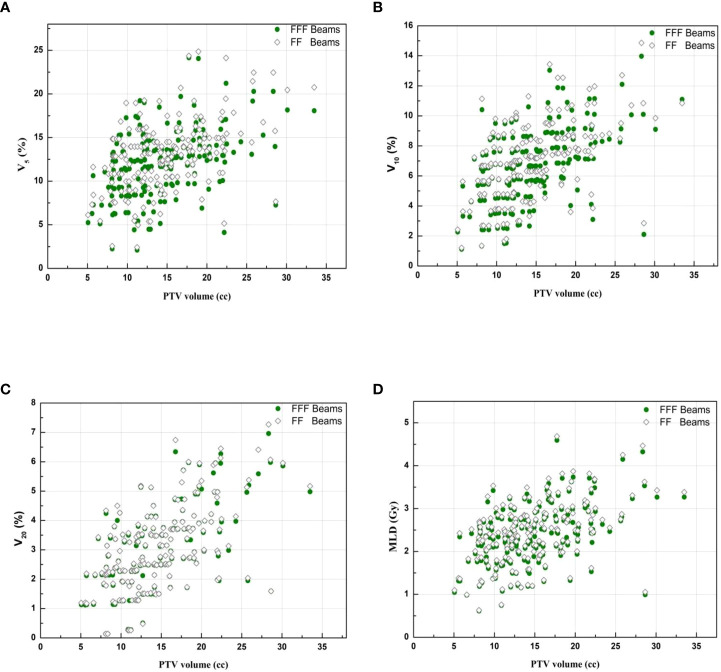
Lung representative dosimetric parameters for FFF beams and FF beams. **(A)**V_5_ of lung. **(B)**V_10_ of lung.**(C)** V_20_ of lung. **(D)** MLD of lung.

All dosimetric parameters of the lung, esophagus, spinal cord and heart were significantly decreased in the VMAT_FFF_ plan (*p*<0.05). The absolute differences in D_1000cc_, D_1500cc_, MLD, V_5_, V_10_, and V_20_of the lung were up to 0.13 Gy, 0.04 Gy, 0.08 Gy, 0.94%, 0.33% and 0.06% higher, respectively, with the VMAT_FF_ plan. Compared with the VMAT_FF_ plan, the VMAT_FFF_ plan provided a lower D_max_, D_0.25cc_ and D_0.5cc_ to the spinal cord, D_max_ and D_5cc_ to the esophagus, and D_1500cc_ to the heart by 0.15 Gy, 0.21 Gy, 0.23 Gy, 0.13 Gy, 0.1 Gy and 0.13 Gy, respectively. No substantial differences were observed in D_max_ and D_4cc_ of the trachea (*p*>0.05). **Figure 2** shows the V_5_, V_10_, V_20_ and MLD of the lung for FFF beams and FF beams for all treatment plans, and the metrics are represented as a function of the PTV volume.

**Figure 3:**
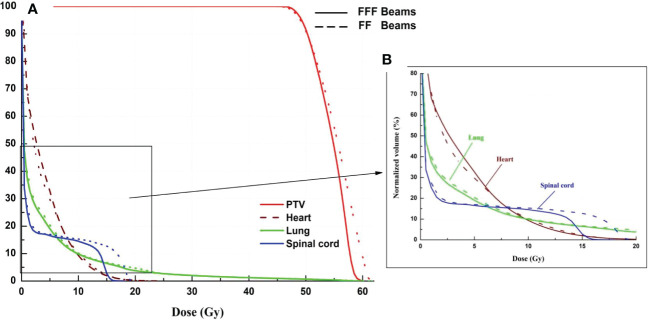
Dose-volume histogram of target and selected OARs. (Solid line: VMAT_FFF_, Dashed line: VMAT_FF_). **(A)** dose ranges from 0 to 61 Gy. **(B)** dose ranges from 0 to 20 Gy.

**Figure 4:**
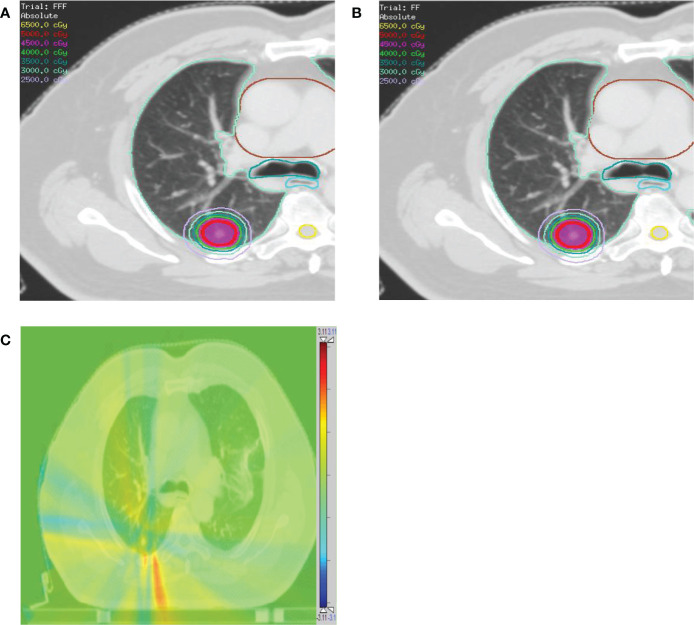
Comparison of dose distributions for a patient with contoured PTV (red), lung (light green), heart (brown), esophagus (cyan), and trachea (dark cyan). **(A)** VMAT_FFF_ plan. **(B)** VMAT_FF_ plan. The dose distribution ranges from 25 Gy to 65 Gy. **(C)** The absolute dose difference distribution between the **(A)** and **(B)**. The dose distribution ranges from -3.11 Gy (blue color) to 3.11 Gy (red color).

**Figure 3** and **Figure 4** show the DVHs and a dose distribution in one patient, respectively. As can be seen in **Figure 1**, for the PTV, the high-dose spillage of the VMAT_FFF_ plan was significantly lower than the VMAT_FF_ plan; for the OAR, the VMAT_FFF_ plan significantly reduced low-dose spillage for the lung and spinal cord. In **Figure 3**, the VMAT_FFF_ plan had the slight advantages of providing tighter intermediate-dose spillage (see R_50%_ and GI) compared with the VMAT_FF_ plan.

### NTCP evaluation of the lung

**Table 5** lists the NTCP values for the VMAT_FFF_ plan and the VMAT_FF_ plan. According to the NTCP model based on MLD, the average values [range] were 1.86 ± 0.19% [1.58%-5.71%] and 1.88 ± 0.19% [1.58%-5.82%] for the VMAT_FFF_ plan and VMAT_FF_ plan, respectively. For the model based on V_5_, the average NTCP [range] value for the VMAT_FFF_ plan was 3.87 ± 1.73% [1.74%-9.01%] compared with 4.17 ± 1.80% [1.83%-9.51%] for the VMAT_FF_ plan. The NTCP based on V_20_ were 3.31 ± 0.70% [2.28%-5.37%] and 3.33 ± 0.73% [2.28%-5.59%] for the VMAT_FFF_ plan and the VMAT_FF_ plan, respectively.

**Table 5:** Comparison of NTCP (mean ± standard)of lung.

Parameters	VMAT_FFF_ (range)	VMAT_FF_ (range)	*p*
MLD (%)	1.86 ± 0.19 (1.58-15.87)	1.88 ± 0.19 (1.58-16.35)	<0.001
V_5_ (%)	3.87 ± 1.73 (1.74-9.01)	4.17 ± 1.80 (1.83-9.51)	<0.001
V_20_ (%)	3.31 ± 0.70 (2.28-5.37)	3.33 ± 0.73 (2.28-5.59)	<0.001

### MUs, beam on-time and treatment time

The average MU values were 1871 ± 278 MUs and 1890 ± 260 MUs for the VMAT_FFF_ plan and the VMAT_FF_ plan, respectively. The average beam on-time was 1.3 times higher for the VMAT_FF_ plan: 4.2 ± 0.3 min, while for the VMAT_FFF_ plan, due to the higher dose rates, the beam on-time reduced to 3.2 ± 0.2 min. The average treatment time were 3.9 ± 0.3 min and 4.8 ± 0.4 min for the VMAT_FFF_ plan and the VMAT_FF_ plan, respectively.

### A volume-based analysis

Patient’s PTVs ranges from 5.07 to 33.49 cc were stratified into four volume groups according to the RTOG 0813 protocol. All values for PTV (R_50%_ and GI), the lung (V_5_, V_10_, V_20_ and MLD) and the NTCP of the lung in the four groups are provided in **Table 6**. The results for the heart, esophagus, trachea and spinal cord are not listed since these OARs were not close to the PTV and do not show a clear trend with the PTV. For all groups, the data showed significant differences between the VMAT_FFF_ plan and the VMAT_FF_ plan (*p*<0.05).

**Table 6:** Summary of the results for parameters of PTV, lung and NTCP (mean ± standard)in four group according to the PTV volume.

	Parameters		Group 1 (range)	Group 2 (range)	Group 3 (range)	Group 4 (range)
PTV	R_50%_	VMAT_FFF_	5.59 ± 0.40 (5.11-6.23)	5.11 ± 0.62 (4.11-6.72)	4.75 ± 0.43 (3.55-5.7)	4.13 ± 0.47 (3.29-5.19)
		VMAT_FF_	5.80 ± 0.45 (5.23-6.42)	5.23 ± 0.63 (4.12-6.88)	4.84 ± 0.46 (3.55-5.79)	4.21 ± 0.49 (3.37-5.44)
	GI	VMAT_FFF_	6.09 ± 0.37 (5.35-6.64)	5.45 ± 0.72 (4.23-6.49)	5.06 ± 0.64 (3.52-6.57)	4.53 ± 0.51 (3.61-5.69)
		VMAT_FF_	6.31 ± 0.37 (5.49-6.68)	5.58 ± 0.75 (4.28-6.83)	5.16 ± 0.67 (3.55-6.91)	4.60 ± 0.59 (3.66-6.61)
Lung	V_5_(%)	VMAT_FFF_	7.55 ± 2.23 (5.11-11.05)	10.86 ± 3.63 (2.11-19.23)	12.95 ± 3.19 (5.14-24.15)	15.03 ± 4.46 (4.12-21.22)
		VMAT_FF_	8.35 ± 2.25 (5.42-11.52)	11.94 ± 3.61 (2.42-19.71)	13.63 ± 3.14 (6.23-24.35)	17.02 ± 5.09 (5.15-24.12)
	V_10_(%)	VMAT_FFF_	3.71 ± 1.67 (1.12-7.12)	5.43 ± 2.90 (1.50-10.41)	6.65 ± 2.26 (3.61-13.04)	7.84 ± 2.83 (2.10-13.97)
		VMAT_FF_	4.04 ± 1.63 (1.21-7.23)	5.76 ± 2.98 (1.66-11.14)	7.07 ± 2.26 (3.32-13.44)	8.58 ± 2.80 (2.85-14.86)
	V_20_(%)	VMAT_FFF_	1.73 ± 0.80 (1.12-3.38)	2.33 ± 1.05 (0.14-5.18)	3.48 ± 1.15 (1.49-6.34)	4.38 ± 1.63 (1.59-6.96)
		VMAT_FF_	1.79 ± 0.80 (1.19-3.43)	2.38 ± 1.07 (0.14-5.18)	3.52 ± 1.17 (1.50-6.74)	4.56 ± 1.72 (1.59-7.27)
	MLD(Gy)	VMAT_FFF_	1.60 ± 0.55 (0.61-2.42)	2.19 ± 0.82 (0.61-3.23)	2.53 ± 0.66 (1.15-4.59)	2.98 ± 0.77 (0.99-4.33)
		VMAT_FF_	1.66 ± 0.57 (0.63-2.52)	2.25 ± 0.83 (0.63-3.27)	2.61 ± 0.66 (1.16-4.68)	3.09 ± 0.77 (1.05-4.47)
NTCP	MLD (%)	VMAT_FFF_	1.86 ± 0.84 (1.79-2.74)	2.44 ± 0.27 (1.58-15.87)	2.91 ± 0.67 (1.83-5.71)	3.38 ± 0.82 (1.74-5.16)
		VMAT_FF_	1.88 ± 0.85 (1.79-2.75)	2.50 ± 0.27 (1.58-16.35)	2.99 ± 0.67 (1.83-5.82)	3.53 ± 0.87 (1.79-5.48)
	V_5_(%)	VMAT_FFF_	2.73 ± 0.50 (2.22-3.51)	3.72 ± 0.79 (1.74-5.37)	3.95 ± 1.02 (2.44-9.01)	4.66 ± 1.28 (2.07-6.94)
		VMAT_FF_	2.92 ± 0.50 (2.33-3.67)	4.05 ± 0.69 (1.83-6.06)	4.16 ± 1.04 (2.62-9.51)	5.33 ± 1.69 (2.28-8.38)
	V_20_(%)	VMAT_FFF_	2.92 ± 0.32 (2.68-3.59)	3.11 ± 0.39 (2.28-8.85)	3.43 ± 0.53 (2.68-4.75)	3.76 ± 0.85 (2.28-5.37)
		VMAT_FF_	2.93 ± 0.33 (2.68-3.59)	3.14 ± 0.38 (2.28-9.18)	3.45 ± 0.55 (2.68-4.75)	3.84 ± 0.91 (2.28-5.59)

p values<0.05 for all parameters.

**Figure 5:**
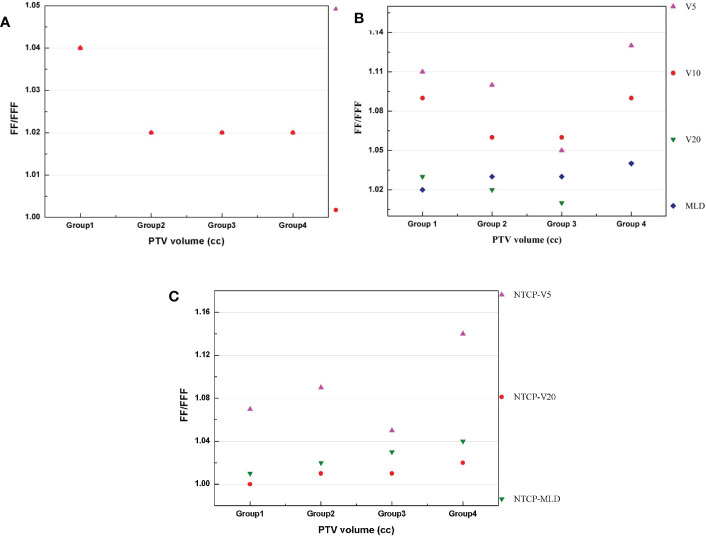
**(A–C)** presents the difference (TD) between two plans for the PTV, lung and NTCP, respectively.

The TD was calculated as follows:


(5) $$\text{TD}=\frac{{\text{V}}_{\text{FF}}}{{\text{V}}_{\text{FFF}}}$$

where V_FF_ and V_FFF_ represent the values for the VMAT_FF_ and VMAT_FFF_ plans, respectively. The GI and R_50%_ values show a decrease between Group 1 and Group 2 which is then stable in Group 3 and 4with increasing volumes. The VMAT_FFF_ plan can be observed as the most advantageous for a small volume in terms of R_50%_ and GI. In Group 1, the TD values for R_50%_ had a mean value of 1.04, while this value for GI was 1.04. In **Figure 5B**, the trend observed was different to that of the GI and the fluctuations were more marked. For V_5_, V_10_, V_20_ and the MLD of lung, the biggest differences between the two plans were in Group 4, at 1.13, 1.09, 1.04 and 1.04, respectively. In **Figure 5C**, the trend observed was similar to that of **Figure 5B** and the biggest difference between the two plans appeared in Group 4.

**Figure 6:**
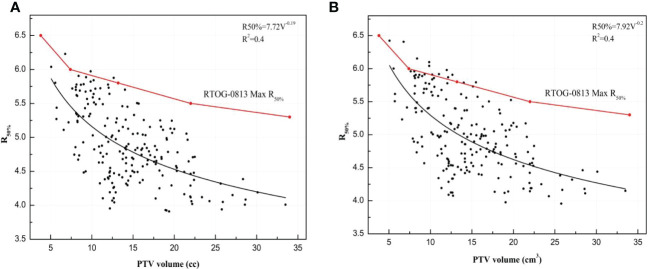
The R_50%_ for VMAT_FFF_ and VMAT_FF_ plans plotted with PTV volume and was presented by a power functional form: **(A)** R_50%_ of VMAT_FFF_ plan. **(B)** R_50%_ of VMAT_FF_ plan.

Furthermore, R_50%_ for the VMAT_FFF_ plan and the VMAT_FF_ plan are described with a power functional form and shown in Eq.(6) and Eq.(7):


(6) $$R_{50\%FFF}=7.72V^{-0.19}$$

where V is the volume of PTV, with a standard error of 0.36 and 0.02 for the A and B parameters, and an R^2^ of 0.4;


(7) $$R_{50\%FF}=7.92V^{-0.2}$$

with an R^2^ of 0.4, and a standard error of 0.38 and 0.02 for the A and B parameters. A plot of R_50%_ versus PTV is presented in **Figure 6**. **Figure 6** shows that VMAT_FFF_ plan have steeply dose fall-off than VMAT_FF_ plans. According to the RTOG 0813/0915 reports, 195 plans exhibited no deviation, 3 plans a minor deviation (less than 10% difference), and none exhibited a major deviation (more than 10% difference) for the VMAT_FFF_ plan; 186 plans exhibited no deviation, 12 plans a minor deviation, and none exhibited a major deviation for the VMAT_FF_ plan.

**Figure 5** The differences between VMAT_FFF_ and VMAT_FF_ plans in four groups: **(A)** R_50%_ and GI of PTV. **(B)** V_5_, V_10_, V_20_ and MLD of lung. **(C)** NTCP of V_5_, V_20_ and MLD.

## Discussion

In the present study, for 175 patients with 198 pulmonary lesions, we investigated the potential advantage of using FFF beams in lung SBRT based on the target volumes. For the investigated VMAT techniques, treatment planning with FFF beams resulted in plans of similar or better quality and a lower NTCP of the lung compared with FF beams. Furthermore, our results indicated that the differences between FFF and FF beams for the parameters of PTV of the lung and the NTCP have been related to the target volume, as shown in **Table 6** and **Figure 5**.

With large doses in a few fractions and a conventional dose rate, the SBRT beam on-time can become very long, increasing patient discomfort and treatment uncertainty. Hence, shortening the SBRT beam on-time becomes crucial. A primary advantage of FFF beams is that they have similar MUs; meanwhile, they shave a shortened beam on-time compared with FF beams. Our results showed that the FFF beams obtained a 23.8% reduction in beam on-time, a 18.8% reduction in treatment time and a 1.0% reduction in MUs for VMAT. A few investigators have reported the time advantage of FFF beams for VMAT plans. For instance, Pokhrel et al. (8) reported that the average beam on-time for FFF beams was 6.5 min, and was much shorter than for FF beams (15.1 min). Pokhrel et al. (15) has shown an overall reduction in beam on-time by about 57.0% using FFF beams for lung lesions. However, in our study, the beam on-time of FFF beams was not significantly reduced compared to the FF beams, which could be related to the DMPO algorithm (Pinnacle) generating segment having less MUs than others TPS. When the dose rate was still increasing, the segment was over. Furthermore, compared to the FF beams, the MUs did not change significantly while using FFF beams. Furthermore, the reason for similar MUs for FFF beams and FF beams is likely to be the small PTV in this study.

One of the major concerns for treating lung cancer using SBRT is the low-dose bath exposure of the lung, such as V_5_, V_10_, V_20_ and the MLD (29). According to the RTOG 0813 recommendation (18), all of the SBRT plans had D_1500cc_<12.5 Gy, D_1000cc_<13.5 Gy and V_20_<10%. The use of FFF beams in lung SBRT treatment has been previously studied (6, 15, 30). Pokhrel et al. (15) designed VMAT plans using FFF beams and FF beams for 13 patients with lung cancer. They found that the VMAT_FFF_ plans had a lower dose to OAR. Similarly, Vassiliev et al. (30) investigated dosimetric differences between FFF beams and FF beams, and reported that FFF beams achieved significantly better target coverage and OAR-sparing than FF beams. This dosimetric advantage of FFF beams was due to the lower out-of-field dose, more rapid dose fall-off, and softer energy spectrum removing the flattening filter compared to FF beams (31). In the current study, utilizing FFF beams for VMAT plans resulted in a better D_2_ and falloff gradient (R_50%_ and GI) and achieved a similar target coverage (D_90_, CR, and CI) compared to FF beam plans. The differences in the D_90_, CR, and CI were not significant. For all plans, the VMAT_FFF_ plans provided similar or better OAR-sparing (lung, esophagus, spinal cord and heart), except the trachea. V_5_, V_10_, and V_20_ of lung were found to be lower by an average of 0.94%, 0.33%, and 0.06% for FFF beams compared with FF beams, respectively. Although, FFF beams has significantly better OAR-sparing than FF beams, the absolute differences between the values are very small. This result might due to the small target volume in our study, the mean volume of PTV is 14.63 ± 5.25 cc. With the target volume increase, the absolute difference between FFF beams and FF beams will increase. For instance, V_5_, V_10_, and V_20_ of lung were lower by an average of 1.99%, 0.74%, and 0.18% for FFF beams compared with FF beams in Group4, respectively.

For the lung, FFF beams appeared the most advantageous for a large-size target (Group 4) in V_5_, V_10_, V_20_ and the MLD of lung. Although the fluctuations were more marked, the trend in the differences increased with an increasing target volume. A different trend was observed for R_50%_ and GI; the biggest differences between two techniques appeared in Group 1: 3.62% for R_50%_ and 3.49% for GI, respectively. There may be several possible explanations for these trends, including (1) there was no significant difference in D_90_, CR, and the CI of PTV between the two techniques due to the profile of the FFF and FFF beams, which are similar; the difference is a lower penumbra, as described by Hrbacek et al. (31) (2); FFF beams have a reduced out-of-field dose that is lower than that for FF beams, especially at the edge of the field and at distances further from the edge; thus, there is a noticeable reduction effect for low doses, such as V_5_ of the lung.

Previous studies have indicated that the dose difference can be significant for radiobiological factors of NTCP (32), which could affect radiotherapy quality. Therefore, evaluating NTCP is a very important part of a comparative study of the two technologies. Timmeren et al. (33) compared a multiple-isocenter technique and a single-isocenter technique for lung SBRT with NTCP based on MLD and V_20_. Kang et al. (34) evaluated the dosimetric and radiobiological parameters in four radiotherapy regimens for synchronous bilateral breast cancer. However, to our knowledge, there are fewer related studies on FFF beams in lung SBRT. Lower doses to the lung of FFF beams resulted in a significantly lower NTCP based on the MLD, V_5_ and V_20_. Evaluated by an NTCP model based on the MLD developed by Borst et al. (26), the risk of radiation pneumonitis decreased, on average, by 0.02% using FFF beams compared to FF beams; this was 0.3% to the model based on V_5_ and 0.02% to the model based on V_20_. We found that lung SBRT with FFF beams could be safely used in order to improve the plan quality and reduce beam on-time. As a result, it is expected that a lower incidence of pneumonitis would be responsible for lung toxicity in lung SBRT patients using FFF beams.

Additionally, we assessed the equations to predict the R_50%_ with a small PTV (<34 cm^3^). Of the 198 lung SBRT plans, 3 exhibited a minor deviation for the VMAT_FFF_ plans and 12 exhibited a minor deviation for the VMAT_FF_ plans, respectively, and none exhibited a major deviation according to the RTOG 0813/0915 reports. It is also worth highlighting that FFF beams have better dose fall-off than FF beams. Hoffman et al. (35) provided the equation of R_50%_ for 317 lung SBRT plans, and the PTV range was from 1.8 cm^3^ to 163 cm^3^. Our equation was larger than Hoffman’s work, which may be due to the values of R_50%_ in our work having a major deviation compared with the equation. The predictive capability of R_50%_ equations for FFF beams and FF beams could function as a useful tool to guide a treatment planner before and after optimization. It can be time-consuming for a planner to generate VMAT plans, and they are often required to generate additional structures such as dose-limiting ring structures. Prior to optimization, the planner could approximately predict the dose distribution of the plan through the R_50%_ equation, and the ring could be generated more accurately, which would reduce the plan optimization time. After optimization, the R_50%_ equation could evaluate the value of R_50%_ with no deviation, minor deviation, or major deviation, and potentially improve the plan quality. As part of the plan quality control (QC), the planner could benchmark the plan against R_50%_ from the equation. Knowing a deviation of R_50%_ compared with the equation, the planner could potentially be spared their efforts in pursuing a better outcome.

However, a limitation of our study was that we only studied a total of 7 and 17 lesions for Group 1 and Group 4, respectively. Future studies are needed to compare the two techniques for more lesions and to increase the PTV range from 34 cc to 95 cc or larger. Moreover, we only selected patients with lung cancer, but the potential for the target and OAR using FFF beams might be suitable for brain and liver SBRT cases. The clinical advantages of FFF beams should be also studied in other tumor cases based on the PTV. Another limitation is that we don’t investigate the interplay effects between the tumor respiratory and dynamic MLC movement for FFF beams and FF beams. The interplay effects plays an important role and could create cold or hot spots inside the lung tumor (36, 37). Fernandez et al. (36) investigated interplay effects in SBRT lung cases and concluded that the interplay effects could result in up to 20% dose variation near the edges of the PTV. However, it has been indicated that the interplay effects causes might negligible dose blurring when using two or more VMAT arcs for FFF beams (38). In the future, one could expand the investigation of interplay effects.

FFF beams and FF beams showed significant differences in dosimetric and radiobiological parameters. For the parameters of the lung, the biggest difference was observed between FFF beams and FF beams for a large target volume; the opposite was observed for the PTV parameter. In this retrospective analysis, we demonstrated that using FFF beams for lung SBRT can reduce the dose to OAR (except the trachea) and potentially provide faster treatment delivery by significantly reducing the beam on-time, when compared with FF beams. Furthermore, using FFF beams could improve patient’s comfort, reduce intra-fraction motion and reduce the probability of radiation pneumonitis. The advantage of FFF beams could be observed with an increasing volume range from 3.8 cm^3^ to 34 cm^3^.

## Data availability statement

The raw data supporting the conclusions of this article will be made available by the authors, without undue reservation.

## Author contributions

Design of the research: JL, FW. Treatment plans: HS. Statistical Analysis: JW, JL. Manuscript preparation: HS, JW. Manuscript writing: HS, JW. Manuscript final revision: BT. All authors contributed to the article and approved the submitted version.

## References

[1] Zheng RS Zhang SW Zeng HM Wang SM Sun K Chen R. Cancer incidence and mortality in China, 2016. J Natl Cancer center (2022) 2(1):1–9. doi: 10.1016/j.jncc.2022.02.002 PMC11256658 39035212

[2] Guckenberger M Andrastschke N Dieckmann K Hoogeman MS Hoyer M Hurkmans C. ESTRO ACROP consensus guideline on implementation and practice of stereotactic body radiotherapy for peripherally located early stage non-small cell lung cancer. Radiother Oncol (2017) 124(1):11–7. doi: 10.1016/j.radonc.2017.05.012 28687397

[3] Postmus PE Kerr KM Oudkerk M Senan S Waller DA Vansteenkiste J. Early and locally advanced non-small-cell lung cancer (NSCLC): ESMO ClinicalPractice guidelines for diagnosis, treatment and follow-up. Ann Oncol (2017) 28(4):iv1–iv21. doi: 10.1093/annonc/mdt241 28881918

[4] Roeber JH Bernhardt D Blanck O Duma M Guckenberger M. Long-term follow-up and patterns of recurrence of patients with oligometastatic NSCLCTreated with pulmonary SBRT. Clin Lung Cancer (2019) 20(6):e667–77. doi: 10.1016/j.cllc.2019.06.024 31327644

[5] Benedict S Yenice K Followill D Galvin JM Hinson W Kavangh B. Stereotactic body radiation therapy: The report of AAPM task group 101. Med Phys (2010) 37(8):4078–100. doi: 10.1118/1.3438081 20879569

[6] Zimmermann F Geinitz H Schill S Grosu A Schratzenstaller U Molls M. Stereotactic hypofractionated radiation therapy for stage I non-small cell lung cancer. Lung Cancer (2005) 48(1):107–14. doi: 10.1016/j.lungcan.2004.10.015 15777977

[7] Al-Hallaq HA Chmura S Salama JK Winter KA Robinson CG Pisansky TM. Rational of technical requirements for NRG-BR001: The first NCI-sponsored trial of SBRT for the treatment of multiple metastases. Pract Radiat Oncol (2016) 6(6):e291–8. doi: 10.1016/j.prro.2016.05.004 PMC5099083 27345129

[8] Pokhrel D Snford L Dhanireddy B Molloy J Randall M McGarry RC. Flattening filter free VMAT for a stereotactic,single-dose of 30 gy to lung lesion in a 15-min treatment slot. J Appl Clin Med Phys (2020) 21(4):6–12. doi: 10.1002/acm2.12829 PMC7170282 32039544

[9] Pokhrel D Visal J Sanford L. A novel and clinically useful dynamic conformal arc (DCA)-based VMAT planning technique for lung SBRT. J Appl Clin Med Phys (2020) 21(7):29–38. doi: 10.1002/acm2.12878 32306530 PMC7386176

[10] Murakami Y Magome T Matsubayashi F Takahashi R Arima M Kamima T. Evaluation of organ-at-risk dose reduction with jaw tracking technique in flattening filter-free beams in lung stereotactic body radiation therapy. Phys Med (2019) 61:70–6. doi: 10.1016/j.ejmp.2019.04.018 31151582

[11] Kragl G Wetterstedt S Knäusl B Lind M McCavana P Knöös T. Dosimetric characteristics of 6 and 10 MV unflattened photon beams. Radiother Oncol (2009) 93(1):141–6. doi: 10.1016/j.radonc.2009.06.008 19592123

[12] Kry SF Vassiliev ON Mohan R. Out-of-field photon dose following removal of the flattening filter from a medical accelerator. Phys Med Biol (2010) 55(8):2155–66. doi: 10.1088/0031-9155/55/8/003 20305334

[13] Aoki S Yamashita H Haga A Nawa K Imae T Takahashi W. Flattening filter-free technique in volumetric modulated arc therapy for lung stereotactic body radiotherapy: A clinical comparison with the flattening filter technique. Oncol Lett (2018) 15:3928–36. doi: 10.3892/ol.2018.7809 PMC5854932 29563993

[14] Hrbacek J Lang S Graydon SN Klöck S Riesterer O. Dosimetric comparison of flattened and unflattened beams for stereotactic ablative radiotherapy of stage I non-small cell lung cancer. Med Phys (2014) 41(3):031709. doi: 10.1118/1.4866231 24593713

[15] Pokhrel D Halfman M Sanford L. FFF-VMAT for SBRT of lung lesions: Improves dose coverage at tumor-lung interface compared to flattened beams. J Appl Clin Med Phys (2020) 21(1):26–35. doi: 10.1002/acm2.12764 PMC6964748 31859456

[16] Tambe NS Fryer A Marsden JE Moore C Beavis AW. Determination of clinically appropriate flattening filter free (FFF) energy using treatment plans and delivery measurements. BioMed Phys Eng Express (2016) 2(6):65016. doi: 10.1088/2057-1976/2/6/065016

[17] Reggiori G Mancosu P Castiglioni S Alongi F Pellegrini C Lobefalo F. Can volumetric modulated arc therapy with flattening filter free beams play a role in stereotactic body radiotherapy for liver lesions? a volume-based analysis. Med Phys (2012) 39(2):1112–8. doi: 10.1118/1.3679858 22320821

[18] Bezjak A Bradley J Gaspar L Timmerman RD Gore E Papiez L. Seamless phase I/II study of stereotactic lung radiotherapy (SBRT) for early stage, centrally located, non-small cell lung cancer (NSCLC) in medically inoperable patients. RTOG (2012) 0813:1–81.

[19] Videtic GM Hu C Singh AK Chang JY Parker W Olivier KR. A randomized phase II study comparing 2 stereotactic body radiation therapy (SBRT) schedules for medically inoperable patients with stage I peripheral non-small cell lung cancer : NRG oncology RTOG 0915 (NCCTG N0927). Int J Radiat Oncol Biol Phys (2015) 93:757–64. doi: 10.1016/j.ijrobp.2015.07.2260 PMC4744654 26530743

[20] Takayama K Nagata Y Negoro Y Mizowaki T Sakamoto T Sakamoto M. Treatment planning of stereotactic radiotherapy for solitary lung tumor. Int J Radiat Oncol Biol Phys (2005) 61(5):1565–71. doi: 10.1016/j.ijrobp.2004.12.066 15817363

[21] Wulf J Hädinger U Oppitz U Thiele W Ness-Dourdoumas R Flentje M. Stereotactic radiotherapy of targets in the lung and liver. Strahlenther Onkol (2001) 177:645–55. doi: 10.1007/PL00002379 11789403

[22] Audet C Poffenbarger BA Chang P Jackson PS Lundahl RE Ryu SI. Evaluation of volumetric modulated arc therapy for cranial radiosurgery using multiple noncoplanar arcs. Med Phys (2011) 38(11):5863–72. doi: 10.1118/1.3641874 22047350

[23] Paddick I. A simple scoring ratio to index the conformity of radiosurgical treatment plans. J Neurosurg (2000) 93(3):219–22. doi: 10.3171/jns.2000.93.supplement_3.0219 11143252

[24] Paddick I Lippitz B. A simple dose gradient measurement tool to complement the conformity index. J Neurosurg (2006) 123:194–201. doi: 10.3171/sup.2006.105.7.194 18503356

[25] Jones B Dale RG Deehan C Hopkins KI Morgan DAL. The role of biologically effective dose (BED) in clinical oncology. Clin Oncol-uk (2001) 13(2):71–81. doi: 10.1053/clon.2001.9221 11373882

[26] Borst GR Ishikawa M Nijkamp J Hauptmann M Shirato H Bengua G. Radiation pneumonitis after hypofractionated radiotherapy: Evaluation of the LQ(L) model and different dose parameters. Int J Radiat Oncol Biol Phys (2010) 77(5):1596–603. doi: 10.1016/j.ijrobp.2009.10.015 20231066

[27] Lyman JT. Complication probability as assessed from dose-volume histograms. Radiat Res Suppl (1985) 104(2):S13–9. doi: 10.2307/3583506 3867079

[28] Seppenwoolde Y Lebesque JV De Jaeger K Boersma LJ Schilstra C Henning GT. Comparing different NTCP models that predict the incidence of radiation pneumonitis. normal tissue complication probability. Int J Radiat Oncol Biol Phys (2003) 55(3):724–35. doi: 10.1016/s0360-3016(02)03986-x 12573760

[29] Baker R Han G Sarangkasiri S Demarco ML Turke C Stevens CW. Clinical and dosimetric predictors of radiation pneumonitis in a large series of patients treated with stereotactic body radiation therapy to the lung. Int J Radiat Oncol Biol Phys (2013) 85(1):190–5. doi: 10.1016/j.ijrobp.2012.03.041 22929858

[30] Vassiliev ON Peterson CB Chang JY Radhe M. Using FFF beams to improve the therapeutic ratio of lung SBRT. J Radiother Pract (2021) 20(4):419–25. doi: 10.1017/S1460396920000576 PMC8939879 35330584

[31] Hebacek J Lang S Klöck S. Commissioning of photon beams of a flattening filter-free linear accelerator and the accuracy of beam modeling using an anisotropic analytical algorithm. Int J Radiat Oncol Biol Phys (2011) 80(4):1228–37. doi: 10.1016/j.ijrobp.2010.09.050 21129855

[32] Srivastava SP Cheng CW Das IJ. The dosimetric and radiobiological impact of calculation grid size on head and neck IMRT. Pract Radiat Oncol (2017) 7(3):209–17. doi: 10.1016/j.prro.2016.10.001 27847266

[33] Timmeren JV Ehrbar S Chamberlain M Mayinger M Hoogeman M Andratschke N. Single-isocenter versus multiple-isocenters for multiple lung metastases: Evaluation of lung dose. Radiother Oncol (2022) 166:189–94. doi: 10.1016/j.radonc.2021.11.030 34864135

[34] Kang SW Kang S Lee B Song C Eom KY Jang BS. Evaluation of the dosimetric and radiobiological parameters in four radiotherapy regimens for synchronous bilateral breast cancer. J Appl Clin Med Phys (2022) 23(8):e13706. doi: 10.1002/acm2.13706 35727562 PMC9359036

[35] Hoffman D Dragojević I Hoisak J Hoopes D Manger R. Lung stereotactic body radiation therapy (SBRT) dose gradient and PTV volume: A retrospective multi-center analysis. Radiat Oncol (2019) 14(1):162. doi: 10.1186/s13014-019-1334-9 31481089 PMC6724320

[36] Fernandez DJ Sick JT Fontenot JD. Interplay effects in highly modulated stereotactic body radiation therapy lung cases treated with volumetric modulated arc therapy. J Appl Clin Med Phys (2020) 21(11):58–69. doi: 10.1002/acm2.13028 33104297 PMC7700928

[37] Kang H Yorke ED Yang J Chui C-S Rosenzweig KE Amols HI. Evaluation of tumor motion effects on dose distribution for hypofractionated intensity-modulated radiotherapy of non-small-cell lung cancer. J Appl Clin Med Phys (2010) 11(3):78–89. doi: 10.1120/jacmp.v11i3.3182 PMC2924766 20717084

[38] Ong CL Dahele M Slotman BJ Verbakel W. Dosimetric impact of the interplay effect during stereotactic lung radiation therapy delivery using flattening filter-free beams and volumetric modulated arc therapy. Int J Radiat Oncol Biol Phys (2013) 86(4):743–8. doi: 10.1016/j.ijrobp.2013.03.038 23773394

